# A Functional Wound Dressing as a Potential Treatment for Cutaneous Leishmaniasis

**DOI:** 10.3390/pharmaceutics11050200

**Published:** 2019-05-01

**Authors:** Francisco Alexandrino-Junior, Kattya Gyselle de Holanda e Silva, Marjorie Caroline Liberato Cavalcanti Freire, Viviane de Oliveira Freitas Lione, Elisama Azevedo Cardoso, Henrique Rodrigues Marcelino, Julieta Genre, Anselmo Gomes de Oliveira, Eryvaldo Sócrates Tabosa do Egito

**Affiliations:** 1 Programa de Pós-Graduação em Nanotecnologia Farmacêutica (PPgNANOFARMA), Universidade Federal do Rio Grande do Norte (UFRN), Nata/RN 59012-570, Brazil; alexandrino_jr@yahoo.com.br; 2 Faculdade de Farmácia, Universidade Federal do Rio de Janeiro (UFRJ), Rio de Janeiro/RJ 21941-902, Brazil; holanda.gyselle@gmail.com (K.G.d.H.eS.); vivianelione@gmail.com (V.d.O.F.L.); elisamaazevedo@gmail.com (E.A.C.); 3 Instituto de Física de São Carlos, Universidade de São Paulo (USP), São Carlos/SP 13563-120, Brazil; marjorie_freire_@hotmail.com; 4 Departamento do Medicamento, Universidade Federal da Bahia (UFBA), Salvador/BA 40170-115, Brazil; henrique.rmarcelino@gmail.com; 5 Faculdade de Farmácia, Universidade Federal do Rio Grande do Norte (UFRN), Nata/RN 59012-570, Brazil; jgenre@gmail.com; 6 Departamento de Fármacos e Medicamentos, Universidade Estadual Paulista (UNESP), Araraquara/SP 14800-903, Brazil; ans_gomes@yahoo.com.br; 7 Laboratório de Sistemas Dispersos (LaSiD), Departamento de Farmácia, Universidade Federal do Rio Grande do Norte (UFRN), Rua General Gustavo Cordeiro de Farias s/n, Petrópolis, Nata/RN 59012-570, Brazil

**Keywords:** Amphotericin B, cutaneous leishmaniasis, hydrogel, wound dressing, controlled release

## Abstract

Cutaneous leishmaniasis (CL) is a parasitic disease characterized by progressive skin sores. Currently, treatments for CL are limited to parenteral administration of the drug, which presents severe adverse effects and low cure rates. Therefore, this study aimed to develop poly(vinyl-alcohol) (PVA) hydrogels containing Amphotericin B (AmB) intended for topical treatment of CL. Hydrogels were evaluated in vitro for their potential to eliminate promastigote forms of *Leishmania* spp., to prevent secondary infections, to maintain appropriate healing conditions, and to offer suitable biocompatibility. AmB was incorporated into the system in its non-crystalline state, allowing it to swell more and faster than the system without the drug. Furthermore, the AmB release profile showed a continuous and controlled behavior following Higuchi´s kinetic model. AmB-loaded-PVA-hydrogels (PVA–AmB) also showed efficient antifungal and leishmanicidal activity, no cytotoxic potential for VERO cells, microbial impermeability and water vapor permeability compatible with the healthy skin’s physiological needs. Indeed, these results revealed the potential of PVA–AmB to prevent secondary infections and to maintain a favorable environment for the healing process. Hence, these results suggest that PVA–AmB could be a suitable and efficient new therapeutic approach for the topical treatment of CL.

## 1. Introduction

Cutaneous leishmaniasis (CL) is a disease caused by a genus of trypanosomatid protozoa called *Leishmania*, transmitted to humans by the bite of infected female phlebotomine sandflies [1]. CL commonly appears first as a localized papule, which evolves into an ulcer upon loss of the epidermis. Afterwards, the impairment of the skin barrier in the lesion leads to the formation of long-life scars and severe skin disabilities. Moreover, CL, which represents the most common form of leishmaniasis, is currently considered a serious public health problem in 98 countries over all five continents [2]. The World Health Organization (WHO) has estimated that 0.7 to 1.3 million new cases of the disease occur worldwide annually [3].

While the development of an effective vaccine against *Leishmania* spp. is still under research [4,5], the use of pentavalent antimony organic compounds (SbV) or pentamidines are the recommended treatment for all leishmaniasis forms [6]. However, their efficiency for getting rid of the parasites is only around 60% [7]. In this context, Amphotericin B [8], an antifungal polyene agent approved by the FDA for clinical use, has been successfully applied when the abovementioned treatments failed, showing in some cases only 15% failure [9]. Nevertheless, the clinical use of AmB is limited due to the severe side effects, mainly nephrotoxicity, that the micelle system formulation containing this drug presents [8]. Besides, the use of AmB requires hospitalization for its intravenous administration, which leads to non-adhesion to the treatment by infected individuals in 75% of cases [7]. On the other hand, when less toxic AmB formulations are used, such as the liposomal ones [10], their cost is prohibitively expensive for people in developing countries, and these dosage forms still require intravenous administration.

Considering the present scenario, the development of new therapeutic approaches to improve CL treatment and to promote its world accessibility is mandatory. In this context, the topical treatment of CL lesions represents an attractive alternative to reduce the systemic toxicity associated with the use of the abovementioned dosage forms administered intravenously, promoting elimination of parasites, re-epithelization of the skin, preventing secondary infections and enabling outpatient treatment.

The currently available local treatments for CL include intralesional injection of SbV [11] or a combination of SbV with physical therapies, such as cryotherapy [12] or thermotherapy [13]. Additionally, the topical administration of paromomycin-methylbenzethonium chloride (PR–MBCL) ointments is an option. However, although this last treatment presents fewer adverse effects, less pain, and easier administration, its therapeutic activity depends on the presence of MBCL in the formulation [14], a cationic surfactant which usually leads to inflammatory reactions. Moreover, Kim et al. demonstrated that PR regimen was less effective than any SbV regimen to achieve a clinical cure, with a described efficacy varying from 17% to 67% for *Leishmania major* infection [14].

Undeniably, the development of topical formulations containing AmB seems to be the ideal approach for CL treatment. However, the commercially available lipid formulations for this drug, when topically applied, are ineffective at curing CL in an animal model [15]. This observation supports the idea that the lack of efficiency of this type of treatment could be a deficiency of the drug delivery rather than a lack of drug efficacy. Therefore, this highlights the need to develop new therapeutic systems loaded with AmB for the topical treatment of CL. 

In this context, the use of hydrogels seems to be a promising strategy, since it combines numerous advantages to wound management, e.g., enhancing the healing process [16,17], reducing pain [18], enabling exchange of gases (e.g., O_2_ and H_2_O) [19], possibility to tailor the mucoadhesion [20], act as a barrier to external threats like microbes [19]. Moreover, it can, simultaneously, act as a carrier for therapeutic agents [21,22,23,24]. 

Hydrogels consist of polymeric networks that absorb large amounts of water while remaining insoluble in aqueous solutions due to chemical or physical cross-linking of their individual polymer chains. Since the first report in 1960, by Wichterle and Lím [25], synthetic hydrogels have been widely applied to biomedical use, and special attention should be given to the ones manufactured with poly(vinyl alcohol) (PVA). Due to its excellent properties (e.g., high biocompatibility, hydrophilicity, transparency, etc.) this polymer has a historical use in biomedical applications [26], e.g., cell culturing [27], artificial cartilage [28], long-term implants [29,30], scaffold for tissue engineering and tissue mimicking [31], wound dressing material [32,33] and soft contact lenses [34]. Lastly, PVA has been used as carriers for therapeutic agents such as drugs [35,36,37,38,39] and as a surface modifier, for liposomes [40,41]. The use of PVA in the pharmaceutical field, as a material to manufacture drug delivery systems, is justified because of its biodegradability [42] and low toxicity [43]. However, due to its high water solubility, PVA needs to be subjected to a cross-linking process in order to manufacture the hydrogel system. To this end, chemical cross-linking is one of the approaches used to enhance its mechanical, chemical and thermal properties [44].

Recently, our group demonstrated the feasibility of PVA hydrogels as a carrier to control AmB release [35]. Thus, the proposal of the present work was to produce an AmB-loaded poly(vinyl alcohol) hydrogel (PVA–AmB) intended to be used as a wound dressing system for CL treatment. With this aim, a 2^3^ full factorial design was applied to evaluate the role of the pH, the degree of cross-linking and the presence of AmB in the system´s constitution. Then, the hydrogel microstructure was characterized by evaluating the AmB aggregation state into the system, surface morphology, swelling degree, drug release kinetics, and water and microbial permeability. The final goal of this proposal was to assess the leishmanicidal, antifungal, and cytotoxic activity of the AmB-loaded system.

## 2. Materials and Methods 

### 2.1. Materials

Glutaraldehyde (GA, 25%*v*/*v* aqueous solution), dimethyl sulfoxide (DMSO) and monobasic anhydrous potassium phosphate were purchased from Quimiobras Indústria Química (Masssaranduba, SC, Brazil). Sodium chloride, potassium chloride, and methanol came from ISOFAR Indústria e Comércio de Produtos Químicos (Duque de Caxias, RJ, Brazil). Amphotericin B came from Indofine Chemical Company (Hillsborough, NJ, USA). Brain heart infusion medium and Sabouraud Dextrose Agar were purchased from HiMedia Laboratories, LLC (Mumbai, India). Streptomycin and penicillin were purchased from LGC Biotecnologia (Cotia, SP, Brazil). Fetal bovine serum (FBS) was purchased from Gibco™ (Gaithersburg, MD, USA). Dibasic sodium phosphate, Schneiders modified medium, Dulbecco´s Modified Eagle´s Medium–High glucose (DMEM–HG), and anhydrous poly(vinyl alcohol) (PVA, 98% hydrolyzed, Mw 13.000–23.000 g/mol) were purchased from Sigma-Aldrich (St. Louis, MO, USA).

### 2.2. Factorial Design for Development of AmB-Loaded Hydrogels

A 2^3^ full factorial design was used to evaluate the influence of three main factors on the system´s characteristics. In this case, the pH (5 and 2), the cross-linker concentration (Glutaraldehyde at 132.5 µM·L^−1^ and at 26.5 µM·L^−1^), and the presence of the AmB in the system (0 and 10 µM) were tested. 

As previously reported, PVA hydrogels were prepared using the casting technique with few modifications [45]. Briefly, PVA powder was accurately weighed, introduced into an airtight container with water, and completely dissolved at 90 °C for 15 min to form a 10%(*w*/*v*) solution. This solution was cooled slowly at room temperature, under magnetic stirring, and the pH was adjusted to 2 with HCl 1 M. Afterward, AmB was added to the PVA solution in a 1:20 ratio (AmB:PVA *w*/*w*), followed by ultrasound bath for 10 min. The cross-linking reaction was carried out for 16 h in a polyethylene dish (diameter of 5.5 cm). Before all analyses, except the X-ray diffraction and the scanning electron microscopy, the hydrogels were immersed twice in distilled water to remove H^+^ excess.

### 2.3. X-ray Diffraction Study 

The X-ray diffraction (XRD) spectrum of the hydrogels with (PVA–AmB) and without AmB (PVA–H), respectively, were carried out in the range from 2° to 80° (2θ) at the speed of 0.05°/s using a diffractometer (Miniflex desktop diffractometer, Rigaku Corporation, Tokyo, Japan). The XRD system was equipped with a CuKα radiation source (λ =1.541 Å, 40 kV and 40 mA). 

### 2.4. Hydrogel Thickness and Morphology

In order to investigate the thickness homogeneity in the PVA–AmB, the systems were virtually divided into two regions, edge and inner. The thickness was determined using a manual ABS digimatic indicator (model 543250B /ID-C112B, Mitutoyo Corporation, Kanagawa, Japan). The final results were expressed as the mean of 20 measurements on the entire film surface (10 at random edge and inner regions, respectively).

The scanning electron micrographs of PVA–H and PVA–AmB were performed using a Scanning Electron Microscope (JSM-5610LV Scanning Electron Microscope, JEOL Ltd, Tokyo, Japan). Prior to evaluation, hydrogels were placed over carbon conductive tabs and sputter-coated with gold plasma. The morphology of the system and the presence of pores were evaluated using an accelerating voltage of 20 kV.

### 2.5. Drug Loading Efficiency (%)

The amount of AmB presented in the system was measured from hydrogel fragments of 1 cm^2^. To this end, these pieces were immersed in 3 mL of DMSO and taken to the ultrasound bath for 15 min. The resulting solution was again diluted in DMSO and the AmB content was measured by spectrophotometry (Libra S32 UV/Vis Spectrophotometer, Biochrom Ltd, Cambridge, UK) at λ = 416 nm. Drug loading efficiency was calculated by the following equation [46]:

(1) $$Drug\;Loading\;\left(\%\right)=\frac{Experimental\;Drug\;Content}{Theoretical\;Drug\;Content}\;\times \;100$$

### 2.6. Determination of Swelling Behavior

A gravimetric approach was used in order to measure the swelling behavior of the system. Therefore, PVA–H and PVA–AmB hydrogels were cut into discs of 1 cm in diameter and immersed into phosphate buffer saline (PBS, pH 7.3 at 25 °C), which was prepared according to the Brazilian Pharmacopoeia [47], for a pre-determined time. Moreover, the samples were taken out of the swelling medium and weighed again after removing PBS excess. The swelling degree (Q) of the hydrogel at each time was calculated as follows [48]:

(2) $$Q=\;\frac{M_{t}-M_{0}}{M_{0}}\;\times \;100$$

where $M_{t}$ and $M_{0}$ are the weight of the system imbibed with the swelling medium at time t and dry sample, respectively.

### 2.7. In Vitro Drug Release 

Since the AmB has a very low water solubility (<1 mg·L^−1^ at pH 6–7) [49], the addition of a solubility enhancer is a usual approach on drug delivery. Therefore, in order to balance the duality of performing changes in the release medium and, at the same time, still produce reliable results, the experiments were performed using the release medium previously reported in the literature [50]. 

The total immersion method was used to evaluate the in vitro AmB release from the system. To this end, hydrogels were cut into fragments of 1 cm^2^ (equivalent to 216 ± 20 µg of AmB) and immersed in 150 mL of release medium (PBS:Methanol (80:20 *v*/*v*)) pH 7.3 at 37 °C, and stirred at 100 rpm. At specified time intervals, aliquots of 3 mL were withdrawn from the solution and the amount of AmB was measured by spectrophotometry (Libra S32 UV/Vis Spectrophotometer, Biochrom Ltd, Cambridge, UK) at λ = 416 nm with a previously validated method. The amount of drug released was calculated and represented as the accumulative percentage of the drug released versus time. To maintain sink conditions, each aliquot was replaced by the same volume of fresh medium.

### 2.8. Mathematical Analysis of the In Vitro Release Kinetics

Different mathematical models were evaluated in order to describe the mechanism of the AmB release that best suited the hydrogels, according to the experimental data obtained from the in vitro release assays (Table 1). Calculations were performed with the support of Add-in DDsolver for Microsoft^®^ Excel [51]. The choice of the model took into account the statistical significance of the fitting and the thermodynamic considerations of the model.

### 2.9. Water Vapor Transmission

The water vapor transmission (WVT) of hydrogels was determined using a modified ASTM E96/E96M water method [52]. Briefly, a test tube containing distilled water was sealed with the hydrogel. To avoid water transport through the edge, the test tubes were thoroughly sealed with scotch tape. Then, the assembly was placed into a chamber at 25 ± 1 °C with a constant relative humidity of 33% ± 1%. Finally, the change in weight of the assembly was measured and the rate of WVT was calculated using the following equation [53]:

(3) $$WVT=\frac{W}{A\times \Delta p}$$

where W is the amount of water vapor permeating through the hydrogel (g·day^−1^), A is the area of exposed hydrogel (cm^−2^), and Δp is the vapor pressure difference (mmHg).

### 2.10. Microbial Permeability Assay

In order to investigate the ability of the hydrogel to prevent microbial penetration, microbiological tests were conducted as previously mentioned [19], with modifications. Briefly, test tubes containing 3 mL of sterile brain heart infusion medium were sealed with sterile hydrogel at aseptic conditions. Positive and negative controls were an open and cap-closed test tube, respectively. The assembly was placed in an open environment and the progress of microbial permeation was observed for 7 days. The cloudiness of the medium in any test tube was recorded as microbial contamination.

### 2.11. Leishmanicidal Activity Assay

In order to estimate the leishmanicidal activity of the PVA–AmB, in vitro assays were performed as previously reported [54] with modifications. Briefly, PVA–H and PVA–AmB hydrogels were cut into fragments of 1 cm^2^ and immersed into Schneiders modified medium. Afterward, late log-phase promastigotes of *Leishmania amazonensis* (IOC/L0575(IFLA/BR/1967/PH8)) and *Leishmania braziliensis* (IOC/L0566(MHOM/BR/1975/M2903) were added to the culture medium in the concentration of 1 × 10^5^ parasites·mL^−1^ and cultured in 24-well plates at 28 °C under 5% CO_2_ for 48 h. At specific time intervals, the promastigote viability was microscopically determined at 400× magnification, through flagellar motility, and counted in a Neubauer chamber. The experiments were carried out under similar conditions using non-treated cells and free AmB (equivalent to a final concentration of 50 µg/mL) as negative and positive controls, respectively.

### 2.12. Antifungal Activity

The disc diffusion approach was used to evaluate the pharmacological efficacy of the PVA–AmB hydrogel against *Candida albicans* (ATCC^®^: 10231), as previously reported [55] with modifications. Briefly, a suspension of this fungal strain, equivalent to 0.5 on the McFarland scale, was homogeneously distributed on a Petri dish with Sabouraud Dextrose Agar using a sterile swab. Afterward, PVA–H and PVA–AmB hydrogels were cut aseptically in fragments of 1 cm^2^ and neutralized with PBS. Then, hydrogels were put on the surface of the culture medium and the zones of growth inhibition were measured after 24 and 48 h of incubation at 28 °C. Control experiments were carried out under similar conditions using 10 µL of an AmB dispersion in PBS (2 mg/mL) as the standard drug with antifungal activity. Microorganism sensitivity to PVA–H and PVA–AmB hydrogels was determined by measuring the size of the inhibitory zones on the agar surface around them.

### 2.13. Cytotoxicity Assay

The nephrotoxicity of the hydrogels was estimated performing an in vitro assay. Firstly, African Green Monkey Kidney (VERO, ATCC^®^ CCL-81) cells were seeded at 10^4^ cells per well and cultured in Dulbecco´s Modified Eagle´s Medium-High glucose (DMEM–HG) supplemented with 10% FBS, streptomycin (100 mg/mL), and penicillin (100 UI/mL). When the cultures reached confluence, transwell filters (0.8 µm; BD) containing PVA–H or PVA–AmB hydrogel fragments of 0.5 cm^2^ (AmB ≈ 113 µg) were applied. Then, the plates were incubated at 37 °C under 5% CO_2_ for 24 h. After treatment, the cytotoxicity was evaluated by the MTT cell proliferation assay [56]. The viability of non-treated control cells was defined as 100%. 

In order to better understand the main effect of AmB toxicity to VERO cell line, a kill curve was performed. To this end, a stock solution of AmB in DMSO was diluted into the culture medium to obtain a concentration of AmB in the range from 5 to 50 µg/mL. The cells were cultivated according to the above-mentioned methodology. 

### 2.14. Statistical Analysis

Statistical analysis was performed by the GraphPad Prism 5.03 software (GraphPad Software Inc, San Diego, CA, USA) using the Shapiro–Wilk test to evaluate the normality of the data distribution, followed by the Bartlett test to assess the homogeneity of variance. Afterward, unpaired *t* tests were performed to determine the difference between the means. The results were presented as the mean of three individual experiments ± standard error of the mean, with *p*-value < 0.05 considered significant.

## 3. Results and Discussion

### 3.1. Factorial Design

**Figure 1:**
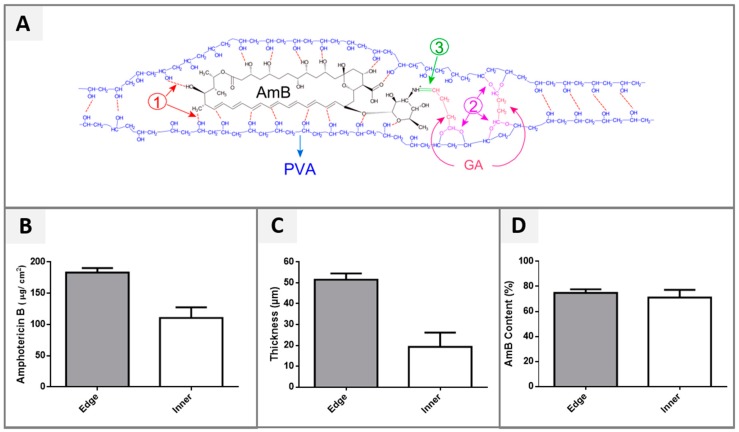
(**A**) Schematic representation of the hydrophilic interactions among the polymer chains, the cross-linker, and the Amphotericin B (AmB). (1) Hydrogen bound, (2) acetal formation and (3) imine formation improving the physical and chemical cross-linking [59]. (**B**) AmB content per unit area (1 cm^2^), (**C**) thickness variation according to the hydrogel region (**D**) AmB values normalized according to the weight of fragments from each respective region.

The influence of the pH over the system’s characteristics was considered significant once only hydrogels produced at pH 2 were able to maintain their structure during the dissolution test in the aqueous medium, while those produced at pH 5 were completely dissolved. Furthermore, the cross-linker concentration also influenced the system´s structure. A synergism during the cross-linking reaction was observed with a higher level of both AmB (at 10 µM) and GA (at 132.5 µM·L^−1^), showing excessive cross-linking bonds, and leading to significant deformations of the hydrogel. This was an unexpected finding and it was ascribed to an increase in the number of hydrophilic interactions among the polymer chains, the cross-linker, and the AmB (Figure 1A), improving the physical and chemical cross-linking occurrence [57,58].

### 3.2. Drug Loading Efficiency (%)

The efficiency of AmB loading into the system was evaluated, as well as a possible difference in the drug distribution between the edge and the inner regions of the hydrogel. In fact, the quantitative analysis of the distribution of the drug into hydrogel showed a significant higher concentration of AmB at the film edges (*p*-value < 0.05), when compared to its inner region (Figure 1B). This result was ascribed to the capillarity phenomenon, which occurred during the cross-linking reaction at the border of the Petri dish used to generate the system, hence increasing the AmB content in this region. 

This hypothesis was corroborated by the thickness comparison and PVA–AmB ratio of both hydrogel regions, inner and edge (Figure 1C). Indeed, the edge region was approximately 2.6 times thicker than the inner region (*p*-value < 0.05), but the PVA–AmB ratio did not change. In fact, the AmB content was normalized according to the weight of each respective fragment and showed no statistical difference between these regions (*p*-value > 0.05, Figure 1D).

Taking into account that the WHO [6] recommends the collection of samples from the swollen edge of CL ulcers for diagnostics due to the highest number of parasites found in this part of the lesion, the heterogeneity observed in the AmB distribution inside the hydrogel can be exploited as a therapeutic strategy. This could assure a better management of the administrated dose, targeting the majority of the active compound to sites where more parasites are found, leading consequently to a more efficient therapy.

### 3.3. Swelling Behavior

The swelling behavior of the hydrogel is a dynamic process, which depends on structural factors such as geometry, chemical composition of the polymeric network, the presence of pores and polymer–solvent interactions [60,61,62,63]. As AmB release from the hydrogel requires the previous solvation of the polymeric chains, swelling kinetic experiments were performed to better understand the mechanisms involved in this process.

**Figure 2:**
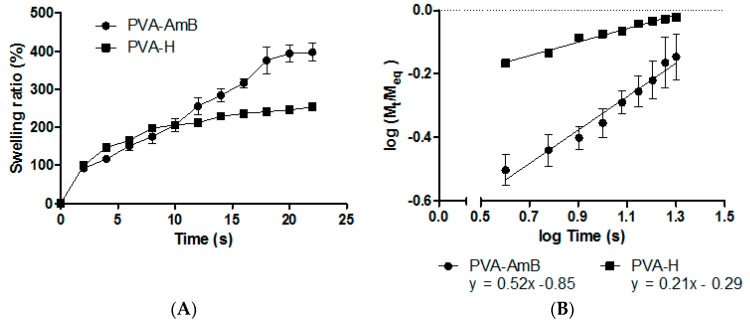
(**A**) Swelling degree of poly(vinyl-alcohol) (PVA)–H and PVA–AmB. (**B**) Log-log plot from which were calculated the diffusional exponent (*n*) and the diffusion constant (*k*) of PVA–H and PVA–AmB in phosphate saline buffer.

Although there was no statistical difference in the swelling behavior of the hydrogels, Figure 2A shows that the PVA–AmB hydrogel had the ability to absorb four times its weight in water. This property could be of great applicability in the treatment of the leishmaniotic ulcers, as it may assist in the removal of secretions, which are presented in up to 86.9% of the lesions [64], and could be extremely beneficial for the healing process.

In addition, Ritger and Peppas [65] showed that from the swelling kinetic assay data it is possible to determine the mechanisms involved in the release process mainly by the use of the following equation:

(4) $$\frac{M_{t}}{M_{\mathrm{eq}}}=kt^{n}$$

where n, k, $M_{t}$ and $M_{\mathrm{eq}}$ represents the diffusional exponent, the diffusion constant, and the mass of the hydrogel at the time t and at the equilibrium, respectively. The values of these constants may be determined by Plotting log($\frac{M_{t}}{M_{\mathrm{eq}}}$) × log(t) (Figure 2B), where the angular and linear coefficients correspond to n and k, respectively [63].

Although the presence of AmB did not change the swelling degree (*p* > 0.05), its presence significantly altered the sorption mechanism. The n values for PVA–H and PVA–AmB hydrogels were 0.2 and 0.52, respectively, which indicate a change in the swelling mechanism, from the Less Fickian to the Limited Relaxation of the polymeric chains one (Anomalous Process) [65]. This hindrance solvent permeation observed for the PVA–H hydrogel was attributed to the strong inter- and intra-chain interactions between PVA and GA in the absence of AmB. This fact can be evidenced by the analysis of the diffusion coefficient values of the solvent into the hydrogels, which were 11.10^−2^ and 17.10^−2^ cm^2^·s^−1^ for PVA–H and PVA–AmB, respectively. Then, the presence of AmB in the hydrogel increased approximately 1.5 times the speed of solvent permeation into the system microstructure.

### 3.4. In Vitro Drug Release

**Figure 3:**
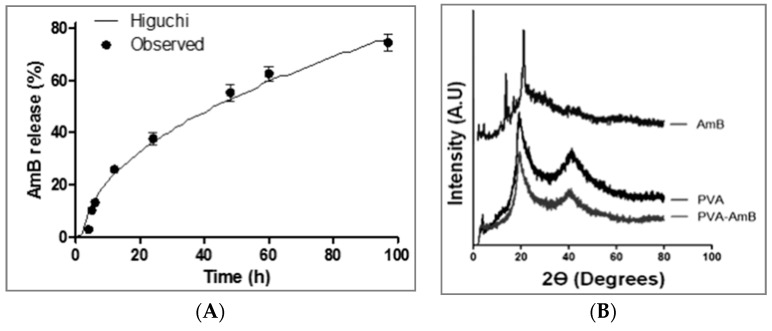
(**A**) Kinetic release of AmB from PVA–AmB hydrogels. Key: (•) experimental data (–) fitted data according to the Higuchi model. (**B**) XRD patterns of the AmB, the PVA–H, and the PVA–AmB hydrogels.

The evaluation of the AmB kinetic release profile from the hydrogel showed that, despite the rapid swelling, the AmB release occurred slowly and gradually. After 97 h, approximately 74% of the total drug content was released from the system. Afterward, the data were fitted using different mathematical models previously described in the literature [51]. The statistical parameters used to compare these different models were the Adjusted Coefficient of Determination (R^2^_adj), which allowed comparing the fitting of the theoretical models to the experimental data, and the Root Mean Square Error (RMSE), which evaluated the difference between the experimental obtained data and the fitted data provided by the used model. Consequently, the model that best fit the experimental data had to exhibit the largest R^2^_adj and the lower RMSE (Table 1). Although from the statistical point of view the Baker–Lonsdale with Tlag was the model that best described the experimental data, from the thermodynamic point of view this model was inappropriate since this approach, adapted from the Higuchi model, is applied to systems having spherical geometry such as microspheres or microcapsules. The literature reports that when the mechanistic models are under investigation, its selection should be based not only on the parameters that fit best, but also on the mechanistic model probability [51]. Thus, the model of Higuchi with Tlag was the chosen model (Figure 3A). This model (Table 1) depicts the release mechanism as a diffusion process based on Fick’s law and dependent on the square root of time.

Another result that corroborates the hypothesis of the AmB release from the hydrogel being governed primarily by diffusion is the system microstructure organization inferred by the XRD analysis (Figure 3B). The diffractogram showed an absence of crystalline peaks ascribed to the AmB, indicating that the AmB is in an amorphous state or molecularly dispersed in the hydrogel. Additionally, the crystallinity index (CI) of the polymeric network calculated through the equation below, was 16.8% and 17.5% for PVA–H and PVA–AmB hydrogels, respectively, and showed no significant changes.

(5) $${\mathrm{CI}}_{\left(\%\right)}=\;\left(1-\frac{A_{a}}{A_{t}}\right)\times \;100$$

where $A_{a}$ and $A_{t}$ are the amorphous phases and the total area, respectively.

Furthermore, the release by erosion mechanisms, as previously reported for PVA hydrogels [66], had not fitted the data, which is justified by the absence of groups subject to hydrolysis in the main chain. Therefore, the AmB release depends only on the solvation, the chain relaxation, and the diffusion.

### 3.5. Water Vapor Permeability

**Figure 4:**
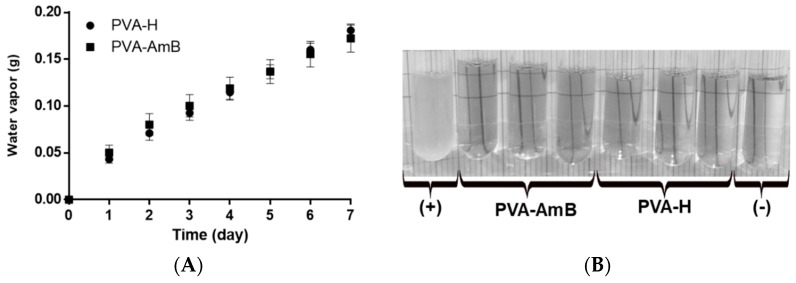
(**A**) Water vapor transmission rate of PVA–H and PVA–AmB hydrogels. (**B**) Medium aspect of positive (+) and negative (−) controls, as well as PVA–H and PVA–AmB after 7 days exposure to the environment, showing the resistance of the hydrogels to microbial permeability.

A key property that needs to be presented by modern wound dressings is the ability to absorb excessive exudates while maintaining a moist environment [67]. Thus, the water vapor transmission of the PVA–H and PVA–AmB hydrogels was evaluated. The results showed that the presence of AmB in the hydrogel network does not significantly affect the water vapor permeability (PVA–AmB = 393 ± 33 g·m^−2^·day^−1^; PVA–H = 452 ± 10 g·m^−2^·day^−1^) (*p*-value > 0.05) (Figure 4A).

The literature reports that the skin in a healthy state has a water vapor permeability of 204 g·m^−2^·day^−1^, while in an injured state it can fluctuate between 279 to 5138 g·m^−2^·day^−1^ [68,69]. Thus, based on our results, it is possible to infer that the PVA–AmB would be able to control the water loss by evaporation from a wound, allowing the skin in an injured state to have a water vapor permeability compatible with that in the healthy state, avoiding dehydration.

### 3.6. Microbial Permeability Assay

The ulcer, the most common clinical manifestation of CL, can remain active for several months, exposing the tissue to microorganisms both from normal skin microbiota and from the environment. The role of the secondary infection in the healing evolution of the leishmaniotic ulcers is still unclear in the literature. However, their acquisition can be considered a grievance to the patient clinical condition [6]. Furthermore, prevention of secondary infection is especially important for *Leishmania*-human immunodeficiency virus (HIV) co-infection individuals, who already have a deficient immunological system [70,71]. Therefore, wound dressing formulations able to treat *Leishmania* infection and avoid secondary microbial infections seem to be of particular importance to these patients.

**Figure 5:**
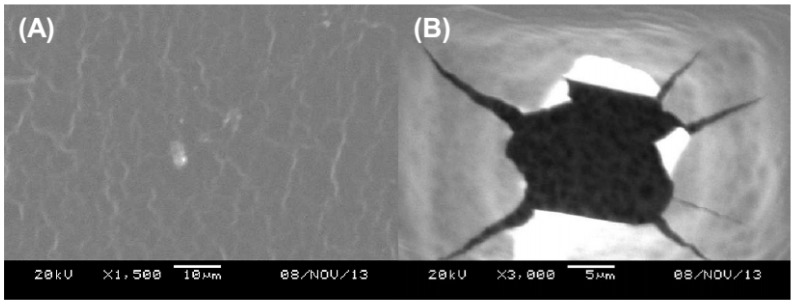
Scanning electron microscope image of PVA–AmB hydrogel evidencing the sample deformation, caused by the electron beam, as the magnification increment goes from 1500× (**A**) to 3000× (**B**).

Thus, the resistance of the hydrogels to microbial permeability was evaluated for 7 days. During the assay, no cloudiness was observed in the culture medium (Figure 4B), which reveals the ability of the hydrogels to act as a barrier for the microorganisms. In fact, this result shows that the use of AmB-loaded hydrogels for treatment of CL may prevent and/or hinder the development of secondary opportunistic infections. As evidenced by the SEM analysis in Figure 5A, the hydrogel displays an apparent nonporous structure. However, it is important to highlight that according to its porosity, the hydrogels are often classified as nonporous/nanoporous (10–100 Å), microporous (100–1000 Å), macroporous (0.1–1 µm) and superporous systems (1–1000 µm) [38,72,73,74]. Since the magnification of 1500× did not provide enough resolution to draw an accurate conclusion about the porosity of the system on a scale smaller than 1 µm (Figure 5A) and, beyond that, a collapse of the hydrogel structure was caused (Figure 5B), no analysis at higher magnification could be performed. Therefore, such classification should be confirmed by further characterization.

Regardless of its classification, either a nonporous system or a microporous one with pores smaller than the pathogens, the system may act as a mechanical barrier. Therefore, the microbial impermeability of the hydrogels produced in this work makes it potentially more efficient in preventing secondary infection than conventional materials such as gauze. [75].

### 3.7. Biological Activity Assays

**Figure 6:**
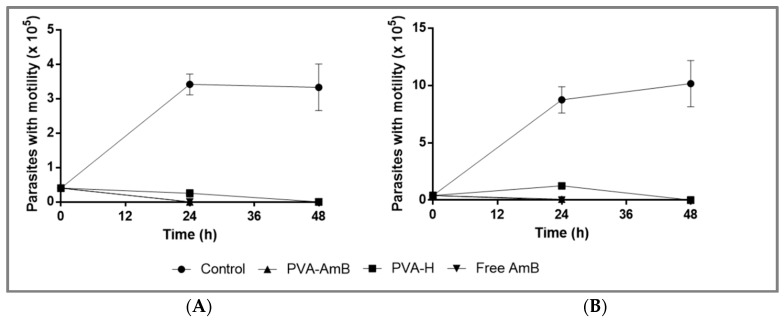
Leishmanicidal activity of the PVA–H and the PVA–AmB against (**A**) *Leishmania amazonensis* and (**B**) *Leishmania braziliensis.*

The leishmanicidal activity of the system against *L. amazonensis* and *L. braziliensis* promastigotes was evaluated for 48 h in a culture medium containing PVA–H and PVA–AmB hydrogels. The results indicated that AmB released from the system performed a similar cytotoxic pattern for *Leishmania* promastigotes (i.e. 100% and 99% ± 2% of mortality for *L. amazonensis* and *L. braziliensis*, respectively) when compared to the positive control, within the first 24 h, evidenced by the absence of flagellar motility of the parasites (Figure 6A,B). Based on the drug release assay (Section 3.4), this is an expected result, since it was expected that the system would release ~38% ± 4% of the initial dose. Therefore, a concentration of AmB equivalent to the positive control (54 ± 6 µg/mL) would be produced.

Zauli-Nascimento et al [56] evaluated the AmB susceptibility of both *Leishmania* species using ATCC strains and clinical strains. The authors reported an AmB effective inhibition concentration of 50% (EC50) ranging from 36 ± 4 to 92 ± 4 ng/mL for the *L. braziliensis* and from 55 ± 1 to 83 ± 6 ng/mL for the *L. amazonensis*. In addition, no significant differences were found among *Leishmania* spp. clinical isolate susceptibility, showing a range of AmB EC90 from 80 to 650 ng/mL.

Thus, considering these findings and assuming that the AmB release in our study had followed the same profile observed in the in vitro drug release assay, the amount of AmB released, within the first 24 h (54 ± 6 µg/mL), would be enough to kill more than 90% of the promastigote forms. Furthermore, considering that the susceptibility of the *Leishmania* promastigote and amastigote forms to AmB was similar, as previously reported [76], it is possible to infer that a similar response to the PVA–AmB observed in vitro for *Leishmania* promastigotes would be observed for the amastigote stage.

Regarding the leishmanicidal activity of the PVA–H, an unexpected decrease in the number of parasites was observed when compared to the negative control (Figure 6A,B). This could be explained by the presence of residual cross-linking inside the polymer matrix, decreasing the promastigote viability. 

**Figure 7:**
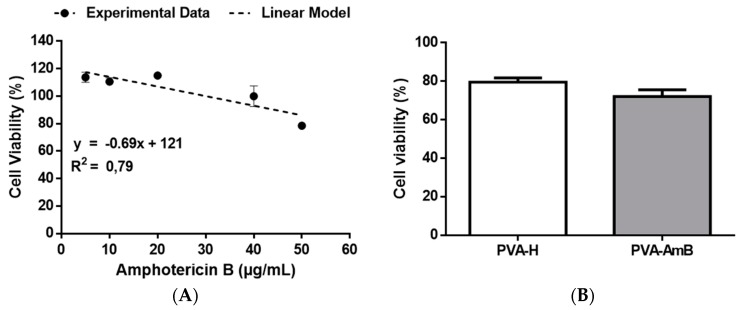
AmB killing curve for VERO cell lines (**A**); evaluation of the cytotoxicity of PVA-H and PVA-AmB against VERO cell lines (**B**).

In order to better understand the AmB cytotoxicity in VERO cells an AmB kill curve was carried out. It seems that the cell viability displays a reasonable linear correlation to the concentration of AmB, as demonstrated by fitting the data through linear regression (Figure 7A). Regarding the cell viability of the hydrogels, no significant difference between PVA–H and PVA–AmB (*p* > 0.05) was observed. 

Performing the same assumption as before, which is the pattern of AmB released into the culture medium follows the one previously observed (Section 3.4), it is possible to compare the cell viability obtained from the hydrogels (Figure 7B, PVA–H = 80% ± 5%; PVA–AmB = 72% ± 7%) to the one predicted by the linear model (~102%). Even though these values of toxicity enable to classify the systems as no cytotoxic potential [77], this analysis revealed that the systems displayed a cytotoxicity 20% higher than expected, which might be related to the presence of residual cross-linking agents.

Furthermore, it is important to highlight that the system here proposed is intended for topical use. Therefore, it is expected that a high dilution factor of the molecules will perhaps reach the bloodstream and, thereafter, the kidneys. For instance, even though the whole dose used on the assay with *Leishmania* spp. (~216 µg) reaches the bloodstream, a dilution by a factor of ~6 L is expected [78]. Therefore, obtaining a concentration of AmB ~36 ng/mL. Taking into account that AmB is highly bound in plasma protein >95% [79], only ~5% will be effectively evaluable to perform the cytotoxic effect. However, no toxic effect is expected with AmB concentrations below 40 µg/mL (Figure 7A). 

Due to the recognized use of AmB as a gold standard on the treatment of fungal infections, mainly the ones caused by *Candida* spp., the antifungal properties of PVA–AmB hydrogels were evaluated by the radial disc diffusion assay against *C. albicans*. The zone of inhibition was measured using free AmB and PVA–H as positive and negative controls, respectively. The PVA–AmB hydrogel resulted in a significant reduction in the viability of *C. albicans*, with inhibitory zones on the agar surface bigger than the positive control (16.7 ± 3 mm vs. 9.8 ± 0.5 mm for 24 h and 16.3 ± 2.5 vs. 9.5 ± 1.3 mm for 48 h, respectively, *p* < 0.05). These results indicate that the AmB entrapped inside of the hydrogel structure keeps its antifungal activity, just as free AmB, while the PVA–H did not show any antifungal activity, corroborating with the idea that AmB stands with its pharmacological activity unaltered and the residual cross-linking quantities were negligible to show antifungal activity or severe cytotoxicity. It has been reported that the affinity of AmB to the ergosterol on fungi membranes is strongly dependent on its aggregation state [80,81]. This phenomenon could be the main reason behind the enhanced antifungal activity of PVA–AmB compared to the free form of the drug. In fact, it is expected that the polymeric matrix of the hydrogel will release the AmB slowly and continuously on its monomeric form. On the other hand, free AmB, which is already evaluable into the medium, should display the aggregated form, since its concentration is higher than 10^−7^ M [82,83,84].

## 4. Conclusions

The results presented here show that the factorial design was a useful tool for the development of PVA hydrogels by the casting method. Parameters like pH, cross-linker, and AmB concentration demonstrated significant effects over the system’s structure. Importantly, the AmB loaded into PVA hydrogels remained in its amorphous state and did not significantly change the crystallinity index. Besides, the PVA–AmB showed water vapor permeation properties that make this kind of system suitable for topical treatment of CL lesions. Furthermore, the applied mathematical modeling showed that the AmB presence in the system altered the swelling mechanism from less Fickian to anomalous, by increasing the hydrogel swelling capacity. However, no burst release of AmB was observed and the release kinetic profile was adequately fitted to the Higuchi model. Additionally, AmB-loaded hydrogels were resistant to microbial permeation and showed effective activity against *Leishmania* promastigotes and *Candida albicans*, as well as no cytotoxic potential for VERO cell lines. These results are indicative of an efficient pharmacological activity and suitable biocompatibility of PVA–AmB hydrogels and show the great potential application of this system for the topical treatment of CL.

## Figures and Tables

**Table 1:** Different mathematical models used for fitting experimental data and their equations with the values of the statistical parameters.

^a^ Model	Equation	R2_adj	RMSE
^b^ Zero-order	$F=\;k_{0}\times t$	0.87	9.8
^c^ First-order	$F=100\;.\;(1-{\;e}^{-k_{1}\times t}$)	0.97	4.7
^d^ Quadratic	$F=100\;\times \left(k_{1}\times \;t^{}+\;k_{2}\times t^{2}\right)$	0.98	4.1
^e^ Higuchi	$F=\;k_{H}\times \;t^{0.5}$	0.93	7.2
^*e^ Higuchi with Tlag	$F=\;k_{H}\times \;{\left(t-\;t_{\mathrm{lag}}\right)}^{0.5}$	0.99	3.1
^f^ Baker–Lonsdale	$\frac{3}{2}\left[1-{\left(1-\;\frac{F}{100}\right)}^{2/3}\right]-\;\frac{F}{100}=\;K_{\mathrm{BL}}\times t$	0.87	9.9
^*f^ Baker–Lonsdale with Tlag	$\frac{3}{2}\left[1-{\left(1-\;\frac{F}{100}\right)}^{2/3}\right]-\;\frac{F}{100}=\;K_{\mathrm{BL}}\times \left(t-T_{\mathrm{lag}}\right)$	0.99	2.3
^g^ Korsmeyer–Peppas	$F=\;k_{\mathrm{KP}}\times \;t^{n}$	0.5	16.5
^*g^ Korsmeyer–Peppas with Tlag	$F=\;k_{\mathrm{KP}}\times {\left(\;t-\;T_{\mathrm{lag}}\right)}^{n}$	0.48	17.7
^h^ Hopfenberg	$F=\;100\times \left[1-\;{\left(1-k_{\mathrm{HB}}\times t\right)}^{n}\right]$	0.94	6.47
^*h^ Hopfenberg with Tlag	$F=\;100\times \left\{1-\left[1-k_{\mathrm{HB}}\times {\left(t-T_{\mathrm{lag}}\right)}^{n}\right]\right\}$	0.94	6.85

^a^ In all mathematic models F represents the fraction (%) of the drug released over time t; ^b^ $k_{0}$ = zero-order release rate constant; ^c^ $k_{1}$ = first-order release rate constant; ^d^ $k_{1}$ = release rate constant for the Quadratic model denoting the dependence of the drug release on the time; *k*_2_ = release rate constant for the Quadratic model denoting the dependence of the drug release on the quadratic time; ^e^ $k_{H}$ = Higuchi release constant; ^f^ $K_{\mathrm{BL}}$ = combined constant on the Baker–Lonsdale model; ^g^ $k_{\mathrm{KP}}$ = release constant incorporating structural and geometric characteristics of the drug-dosage form; n = diffusional exponent indicating the drug-release mechanism; ^h^ $k_{\mathrm{HB}}$ = combined constant on Hopfenberg model; n = 1, 2, and 3 for a slab, cylinder, and sphere, respectively; * $T_{\mathrm{lag}}$ = lag time prior to drug release.
